# The TRPM2 ion channel regulates metabolic and thermogenic adaptations in adipose tissue of cold-exposed mice

**DOI:** 10.3389/fendo.2023.1251351

**Published:** 2024-02-06

**Authors:** Andrea Benzi, Markus Heine, Sonia Spinelli, Annalisa Salis, Anna Worthmann, Björn Diercks, Cecilia Astigiano, Raúl Pérez Mato, Adela Memushaj, Laura Sturla, Valerio Vellone, Gianluca Damonte, Michelle Y. Jaeckstein, Friedrich Koch-Nolte, Hans-Willi Mittrücker, Andreas H. Guse, Antonio De Flora, Joerg Heeren, Santina Bruzzone

**Affiliations:** ^1^ Department of Experimental Medicine-Section of Biochemistry, University of Genova, Genova, Italy; ^2^ Department of Biochemistry and Molecular Cell Biology, University Medical Center Hamburg-Eppendorf, Hamburg, Germany; ^3^ Laboratory of Molecular Nephrology, IRCCS Istituto Giannina Gaslini, Genova, Italy; ^4^ Department of Surgical Sciences and Integrated Diagnostics (DISC), University of Genoa, Genova, Italy; ^5^ Pathology Unit, IRCCS Istituto Giannina Gaslini, Genova, Italy; ^6^ Institute of Immunology, University Medical Center Hamburg-Eppendorf, Hamburg, Germany; ^7^ IRCCS Ospedale Policlinico San Martino, Genova, Italy

**Keywords:** TRPM2, ADPr, white adipose tissue, brown adipose tissue, thermogenesis, cold exposure, browning

## Abstract

**Introduction:**

During thermogenesis, adipose tissue (AT) becomes more active and enhances oxidative metabolism. The promotion of this process in white AT (WAT) is called “browning” and, together with the brown AT (BAT) activation, is considered as a promising approach to counteract obesity and metabolic diseases. Transient receptor potential cation channel, subfamily M, member 2 (TRPM2), is an ion channel that allows extracellular Ca^2+^ influx into the cytosol, and is gated by adenosine diphosphate ribose (ADPR), produced from NAD^+^ degradation. The aim of this study was to investigate the relevance of TRPM2 in the regulation of energy metabolism in BAT, WAT, and liver during thermogenesis.

**Methods:**

Wild type (WT) and *Trpm2^-/-^* mice were exposed to 6°C and BAT, WAT and liver were collected to evaluate mRNA, protein levels and ADPR content. Furthermore, O_2_ consumption, CO_2_ production and energy expenditure were measured in these mice upon thermogenic stimulation. Finally, the effect of the pharmacological inhibition of TRPM2 was assessed in primary adipocytes, evaluating the response upon stimulation with the β-adrenergic receptor agonist CL316,243.

**Results:**

*Trpm2^-/-^* mice displayed lower expression of browning markers in AT and lower energy expenditure in response to thermogenic stimulus, compared to WT animals. *Trpm2* gene overexpression was observed in WAT, BAT and liver upon cold exposure. In addition, ADPR levels and mono/poly-ADPR hydrolases expression were higher in mice exposed to cold, compared to control mice, likely mediating ADPR generation.

**Discussion:**

Our data indicate TRPM2 as a fundamental player in BAT activation and WAT browning. TRPM2 agonists may represent new pharmacological strategies to fight obesity.

## 1 Introduction

Adipose tissue is important for energy balance, glucose and lipid homeostasis and thermogenesis. Excessive nutrient-derived energy is mainly stored in white adipose tissue (WAT) and leads to the expansion of total body mass. Obesity is a consequence of altered energy balance and develops when energy intake exceeds total energy expenditure, which is dictated by the basal metabolic rate, physical activity, and thermogenesis. Obese subjects are at high risk for developing complications such as type 2 diabetes (T2D), cardiovascular disease, and the metabolic syndrome (1). Brown adipose tissue (BAT) is the second main type of adipose tissue. The brown adipose cell contains multilocular small lipid droplets and a high number of mitochondria. Indeed, BAT does not store lipids as much as it oxidizes them. Thus, at least in rodents, BAT is one of the major thermogenic sites of the organism, expressing Uncoupling Protein 1 (UCP1), the protein proton carrier needed for heat production (2). BAT activation is promoted by a number of stimuli, including norepinephrine, thyroid hormones, fibroblast growth factor 21 (FGF21) and bile acids (2–6).

Beige adipocytes, sharing metabolic and morphologic features both with white and brown adipocytes (7), increase during the so-called WAT “browning”, which is triggered by the same stimuli needed to promote thermogenesis in BAT (6). However, induction of browning in WAT is reversible, thus the cells can differentiate again to white adipocytes. To counteract T2D and other obesity-related metabolic disorders, activation of thermogenic activity in WAT and/or BAT has been shown to protect from diet-induced obesity and insulin resistance in many rodent models (8, 9). A number of studies suggest that browning might represent a promising strategy to alleviate metabolic disturbances also in humans (9): (i) BAT activity improves insulin sensitivity (10) and has a negative correlation with BMI (11); (ii) beige adipocytes in WAT are essential to maintain whole-body metabolic homeostasis during catabolic conditions (12, 13); (iii) subjects with an active BAT exhibit improved metabolic health compared with subjects with reduced BAT (14, 15). At present, there are no browning-inducing methods that could be appropriate as therapies and the identification of mechanisms, able to induce long-lasting WAT browning, is highly demanded (9, 15, 16).

The transient receptor potential melastatin 2 (TRPM2) is a nonvoltage-activated ion channel that mediates influx of monovalent and divalent cations including Ca^2+^ from the extracellular space towards the cytosol. The main agonists discovered so far are ADPR, the ADPR-related molecule 2′-deoxyadenosine 5′-diphosphoribose (2dADPR), and H_2_O_2_ (17, 18). It is still unclear whether TRPM2 activation by H_2_O_2_ is direct or indirect (19–21), but it was shown that H_2_O_2_ is able to evoke a rise in 2dADPR levels, which subsequently induce a TRPM2-mediated calcium response in Jurkat T cells (18). Beside these three agonists, cyclic ADPR (cADPR) and NAADP have been described to act as TRPM2 activators, although with only a weak and controversial effect. A recent study seems to conclusively exclude the possibility that cADPR can act as an agonist for TRPM2 (22).

In the context of metabolism and thermogenesis, TRPM2 has not been studied in detail. For instance, TRPM2 was found to be essential in insulin release from pancreatic β-cells. Insulin release requires intracellular Ca^2+^ increase, that involves ATP-sensitive K^+^ channels and TRPM2. Thus, insulin release is hampered in pancreas lacking TRPM2 expression, causing hyperglycemia and T2D (23). Conversely, another study showed that TRPM2 deletion caused a higher glucose metabolism in peripheral tissues, which was associated with higher insulin sensitivity. In addition, mice lacking TRPM2 displayed protection to diet-induced obesity, that was explained by a higher energy expenditure and a lower adipose tissue inflammation in *Trpm2^-/-^* compared to wild type (WT) controls (24). Moreover, pharmacological TRPM2 inhibition was shown to improve insulin sensitivity in adipose tissue during angiotensin II-induced hypertension, suggesting that targeting TRPM2 may be a novel therapeutic strategy to treat hypertension-associated insulin resistance (25). Thus, from the literature, TRPM2 can likely play distinct roles, by regulating both the endocrine system and metabolism in peripheral tissues. Regarding liver, TRPM2 has been shown to be relevant in oxidative stress conditions (26) and in hepatic Ischemia-Reperfusion injury (27). However, as mentioned above, the role played by TRPM2 in adipogenesis and thermogenic response has not been established yet; nevertheless, TRPM2 expression in both BAT and WAT has been documented (28). More data on browning and BAT activation are available regarding the involvement of another member of the TRP channels, TRPM8, expressed by WAT and BAT and representing the cold-sensing receptor in these cells, whose activation induces a rise in the intracellular Ca^2+^ concentration ([Ca^2+^]_i_) and UCP1 expression (29).

Given that the multifunctional ectoenzyme CD38 utilizes NAD^+^ to produce TRPM2 ligands and based on our observation that CD38 is downregulated in activated BAT (30), we aimed to investigate whether the CD38-TRPM2 axis is modulated during cold-induced BAT activation and WAT browning. Our results demonstrate that CD38 and TRPM2 are modulated in opposite directions during thermogenesis and that TRPM2 is involved in adaptive thermogenic responses in BAT and WAT.

## 2 Materials and methods

### 2.1 Materials

CL316,243 (CL) was obtained from Adipogen Life Sciences, San Diego, CA; Flufenamic acid (FFA), Clotrimazole (CLOT) and insulin were from Merk Millipore, Burlington, MA; rosiglitazone was purchased from MedChemExpress, Monmouth Junction, NJ.

### 2.2 *In vivo* experiments

All *in vivo* experiments were conducted in accordance with the laws and institutional guidelines for animal care and were performed with permission of the Animal Welfare Officers at University Medical Center Hamburg-Eppendorf. Mice were housed in temperature- and light-controlled conditions (22°C, 12-h light cycle) with food and water *ad libitum*. *Trpm2^-/-^* mice were kindly provided by Dr. Yasuo Mori (31) and *Cd38^-/-^* mice by Prof Francis Lund (The University of Alabama at Birmingham, Birmingham, AL). Three months old male WT, *Cd38^-/-^* and *Trpm2^-/-^* mice were used for this study (7–8 animals/group). Three groups (the two knockout and WT mice, used as control) were kept for 7 days at 30°C; three groups were kept at 22°C and for the last 24 h at 6°C. Mice were fasted during the last 4 h before the end of the experiments and were euthanized: interscapular BAT (iBAT), inguinal WAT (iWAT) and liver were collected, and flash frozen in liquid nitrogen for future investigations.

### 2.3 qPCR analyses

RNA was isolated from tissues using peqGOLD TriFast (Peqlab), by homogenizing tissues with TissueLyser (Qiagen, Milan, Italy) and purifying RNA by NucleoSpin RNAII Kit (Macherey-Nagel). Afterwards, cDNA was prepared with High-Capacity cDNA Archive Kit (Applied Biosystems). The cDNA was used as template for real-time PCR analysis: reactions were performed in an iQ5 real-time PCR detection system (Bio-Rad) following the experimental conditions described before (32). The used specific primers were described in (30, 33). The following primers were designed through Beacon Designer 2.0 Software (Bio-Rad): *Trpm2* (fw 5’-CCAATCTCCGACGAAGCAATAGC-3’; rv 5’-CATATTGGTGTGCGTGTGTGATGG-3’); *MacroD1* (fw 5’-TGAGCACCTCCACCGACT-3’, rv 5’-TGTTCCTCTCTCTGCTTGTCAC-3’) *MacroD2* (fw 5’-AGCTGAAGTCCACAAAGATGAA-3’, rv 5’-TTGGGAAGTTCTTGGTCACACA-3’); *Oard1* (fw 5’-TGGAGGCCATGAAGTCCCAT-3’; rv 5’-CCGATCCAGACCACATCCAA-3’); *Adprs* (fw 5’-TCCTGAGTCACGTCGAGAGC-3’, rv 5’-TGGCAGTGTCATCTGTGTAGT-3’); *MT-ND1* (fw 5’-CCACGCTTCCGTTACGATCA-3’, rv 5’-GTATGGTGGTACTCCCGCTG-3’). The primers for the housekeeping genes were as follows: *Tbp* (fw 5’-GAAGCTGCGGTACAATTCCAG-3’, rv 5’-CCCCTTGTACCCTTCACCAAT-3’); *Hprt1* (fw CCCTGGTTAAGCATACAGCCCC-3’, rv AGTCTGGCCTGTATCCAACACTTCG-3’).

Total DNA was extracted from iWAT, iBAT and liver of mice (approximately 10 mg) using the QIAamp DNA Micro Kit (Qiagen), according to the manufacturer’s protocol. The purity and quantity of DNA were evaluated with the NanoDrop 1000 (Thermo Fisher Scientific, Waltham, MA). The mitochondrial/genomic DNA ratio was determined using specific primers for mitochondrial tRNALeu (MT-DNA) and genomic/nuclear *Hprt1* by qPCR analysis. The following primers were used: *MT-DNA* (fw 5’-CCGTCACCCTCCTCAAATTA-3’, rv 5’-GGGCTAGGATTAGTTCAGAGTG-3’); *Hprt1* (fw 5’-ATGAAGGAGATGGGAGGCCA-3’, rv 5’-TCCAGCAGGTCAGCAAAGAA-3’).

Statistical analysis of the qPCR was performed using the iQ5 Optical System Software version 1.0 (Bio-Rad) based on the 2^−ΔΔ^Ct method (34). The dissociation curve for each amplification was analyzed to confirm absence of not specific PCR products.

### 2.4 Western blot analysis

Samples from iBAT and iWAT for Western blot analyses were prepared as in (30). Tissue homogenates (30 μg of protein) were loaded on a 10% polyacrylamide gel and proteins were separated by SDS-PAGE and transferred to nitrocellulose membranes. Detection was performed using primary antibodies (anti-Vinculin and anti-Akt, Cell Signaling Technology, Danvers, MA; anti-UCP1, Novus biologicals, Centennial, CO; anti-Poly ADP-ribose, Merck Millipore; anti-phospho-Akt, Santa Cruz Biotechnology, Dallas, TX), following incubation with the appropriate Horseradish Peroxidase (HRP)-conjugated secondary antibodies and Enhanced Chemiluminescence (ECL) detection (GE Healthcare, Milan, Italy). Band intensity was quantified with the ChemiDoc imaging system (Bio-Rad, Milan, Italy).

### 2.5 Immunohistochemistry and histology

For immunohistochemistry, sections of paraffin-embedded adipose tissues were mounted on Superfrost™ Plus adhesion slides, then deparaffinized and rehydrated. Antigen retrieval was performed by boiling the slides in citrate buffer (10 mM sodium citrate, 0.05% Tween 20, pH 6) in a water bath for 30 min. Slides were rinsed with PBS and incubated with blocking buffer (3% BSA in PBS) for 1 h at room temperature in a humidity chamber. The slides were incubated with an antibody detecting UCP1 (ab10983, Abcam) diluted 1:1000 in 1% BSA in PBS overnight at 4°C. After primary antibody incubation, the slides were washed with PBS for 3x 10 min and then incubated with anti-rabbit Cy3 secondary antibody (711-166-152, Jackson) at a 1:500 dilution for 1 h at room temperature. For histology, adipose tissues were fixed in 3.6% formalin in phosphate-buffered saline. Hematoxylin/Eosin (HE) staining was performed on sections of paraffin-embedded tissues using standard procedures. Images were taken using a NikonA1 Ti microscope equipped with a DS-Fi-U3 brightfield camera.

### 2.6 Isolation of mature brown adipocytes from cold-exposed mice

Mature brown adipocytes were obtained from cold-exposed or control WT mice, as described in (30).

### 2.7 Mass spectrometry

BAT and WAT samples from WT and *Cd38^-/-^* mice were deproteinized by mincing them in 0.6 M PCA. Mature brown adipocytes, isolated as in Section 2.6, were incubated with 1 μM CL316,243 for 4 h in Hank’s Balanced Salt Solution. At the end of the incubation, cells were sonicated: one aliquot was used for protein content determination, and 0.6 M PCA was added to the remaining part. The acidic extracts were neutralized with 2 M K_2_CO_3_ and ADPR quantification was performed by HPLC/MS, using a reverse-phase XBridge C18 column (Waters, 150 × 1 mm, 3 μm). Standards and samples were analyzed using a mobile phase consisting of water/methanol (95:5, v/v), containing 4 mM dibutylamine acetate (eluent A) and water/acetonitrile (25:75, v/v; eluent B). The mobile phase started with 0% B, and then increased to 80% B over 10 min and finally to 100% over the next 5 min. The mobile phase was held at 100% B for 5 min and then re-equilibrated to 0% B for 15 min. The flow rate was 30 μl/min. A diverter valve was employed to reduce the introduction of matrix components in the spectrometer. The mass spectra were acquired using electrospray ionization in negative-ion mode in the 100–800 m/z range, and ion charged control with a target ion value of 100,000 and an accumulation time of 300 ms. MS and MS/MS were used for the specific detection of each analyte. The settings of the ESI source were as follows: capillary voltage of 3300 V, nebulizer pressure of 15 psi, drying gas of 8 l/min, dry temperature of 325°C, and 2 rolling averages (averages: 5) were the parameters set for the MS detection. MS/MS analysis was conducted using an amplitude optimized time by time for each compound (35).

### 2.8 Isolation, culture and differentiation of primary adipocytes

Samples of iBAT and iWAT, collected from WT mice, were used to isolate cells from the stromal vascular fraction (SVF) as described (36). In brief, collected AT pieces were washed with 0.9% NaCl solution, minced using scissors and digested at 37°C for 30 min (WAT) or 40 min (BAT) using an “isolation buffer” (123 mM NaCl, 5 mM KCl, 1.3 mM CaCl_2_, 5 mM glucose, 100 mM HEPES, pH 7.4) with the addition, immediately before use, of collagenase II (600 U/ml) and BSA 1.5%. The obtained preparations were passed through a sterile 100 µm filter in a falcon tube, kept on ice for 30 min; subsequently, the middle layer was collected, while the lower layer (debris of tissue) and the upper one (mature adipocytes) were discarded. A second filtration was performed using a 40 µm filter and the product was centrifuged for 10 min at 800x*g*: the supernatant was discarded and the pellet was resuspended in culture medium (DMEM Glutamax-I). Cells were cultured at 37°C, in a humidified atmosphere containing 5% CO_2_ and were differentiated by adding fetal bovine serum (FBS)/newborn calf serum (NCS) (10%), Pen/Strep (1%), Anti-Anti (1%), insulin (2.4 nM), and rosiglitazone (1 μM for brown adipocytes, 100 nM for white/beige adipocytes).

### 2.9 Ca^2+^ imaging in primary brown adipocytes of WT and *Trpm2^-/-^* mice

Primary brown adipocytes from WT and *Trpm2^-/-^* mice were isolated from iBAT as described in 2.8. For Ca^2+^ measurements, isolated cells were seeded in culture medium (DMEM Glutamax-I) on glass bottom Microwell dishes (Mattek P35G-0-20-C) and differentiated in these dishes for 9 days by adding FBS/NCS (10%), Pen/Strep (1%), Anti-Anti (1%), insulin (2.4 nM), and rosiglitazone (1 µM). Medium was replaced every other day and cells were cultured at 37°C, in a humidified atmosphere containing 5% CO_2_. On the day of Ca^2+^ measurements, culture medium was removed and brown adipocytes were incubated in RPMI medium containing 4 μM Fura2-AM for 30 min at 37°C, during which 4 mL of fresh medium were added after 15 min of incubation. Afterwards, cells were carefully rinsed twice and resuspended in Ca^2+^ buffer (140 mM NaCl, 5 mM KCl, 1 mM MgCl_2_, 1 mM CaCl_2_·6H_2_O, 20 mM Hepes, pH 7.4, 1 mM NaCl, 5 mM glucose). The Fura2-loaded adipocytes were imaged on a Leica IRBE microscope using a 40× objective, frames were acquired with an electron-multiplying charge-coupled device camera (EM-CCD; C9100-13, Hamamatsu), and a Sutter DG-4 with the following filter set was used as a light source: excitation (ex), HC 340/26 nm and HC 387/11 nm; beam splitter (bs), 400DCLP; emission (em), 510/84 nm. At 1 min after the start of acquisition, 10 µL Ca^2+^ buffer was added as negative control and the stimulation was achieved by adding 1 µM CL after 2 min. The free cytosolic Ca^2+^ concentration ([Ca^2+^]_i_), was calculated by the Grynkiewicz equation. In brief, R_min_ [using the lowest ratio (R) and fluorescence (F) after EGTA chelation] and R_max_ [using the highest R and F after Ionomycin incubation] of Fura2 in single-cell measurements were determined. The area under the curve was determined with a baseline of 214 nM Ca^2+^ and the mean peak was calculated using the timepoint of the mean maximum peak (WT = 196 s; *Trpm2^-/-^* = 158 s).

### 2.10 Ca^2+^ measurements in primary hepatocytes

Hepatocytes were isolated from WT mice, following the protocol described in (37). Briefly, liver was dissociated in digestion buffer (DMEM high glucose, 1% Penicillin/Streptomycin, 1 mg/mL Collagenase IV, 10 µg/mL HEPES and 10% FBS) and washed once in plating media (DMEM high glucose, 2 mM Glutamax, 2% Penicillin/Streptomycin, 2 mM Sodium Pyruvate, 1 µM Dexamethasone, 0.1 mM Insulin and 10% FBS). The homogenate was passed through a 70 µm strainer and the resulting cell suspension was centrifugated at 50xg for 5 min. To eliminate the red blood cells, an additional centrifuge (20xg for 3 min) was performed and the remaining supernatant was removed. Hepatocytes were then isolated from the non-viable and parenchymal cells using a 90% Percoll (GE healthcare) solution. Cell suspension was centrifuged at 1000xg for 10 min and washed twice with plating media to remove Percoll. Isolated hepatocytes were counted using trypan blue and seeded in poly-lysine-coated, glass bottom cell culture dishes (Greiner Bio-One, Frickenhausen, Germany). Four hours later, the plating medium was replaced with complete medium (DMEM high glucose, 2 mM Glutamax, 2% Penicillin/Streptomycin, 2 mM Sodium Pyruvate, 1 µM Dexamethasone, 0.1 mM Insulin and 0.2% BSA). After 18 hours, hepatocytes were incubated in complete medium with 10 μM Fura2-AM for 40 min at 37°C and washed with Hanks’ balanced salt solution (HBSS). [Ca^2+^]_i_ measurements and calibrations (using the Grynkiewicz equation) were performed with a microfluorimetric system (Cairn Research, Faversham, Kent, UK).

### 2.11 Indirect calorimetry and body temperature measurements

Indirect calorimetric measurements were performed using a Promethion Sable System. WT and *Trpm2^-/-^* mice were transferred to the measurement chamber in new cages without additional nesting material one day before the measurements started. During the experiments, mice were maintained at a 12-h light–dark rhythm with free access to a standard chow diet and water. Oxygen and carbon dioxide levels in each cage were measured every 15 min. Energy expenditure (EE) was calculated using a modified Weir equation (38). For gradual cold exposure studies, mice were acclimated at 30°C and gradually exposed to 5°C, for 6 days starting at 7:00 am. At the end of the experiment, mice were acclimated at 30°C for 24 h and then injected with CL316,243 (1 mg/kg body weight).

### 2.12 Statistical analyses

Groups were compared by an unpaired Student’s t test, or by one way ANOVA followed by Tukey’s Test, using GraphPad software. Values of p < 0.05 were considered significant.

## 3 Results

### 3.1 *Trpm2^-/-^* mice display reduced upregulation of thermogenic markers in WAT and BAT after cold exposure

**Figure 1:**
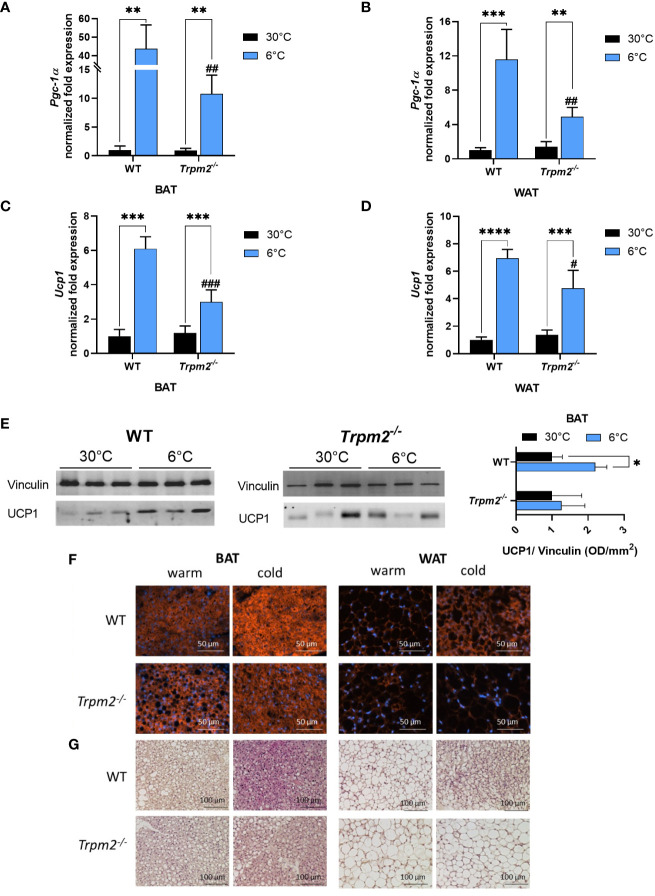
Lack of TRPM2 impairs cold exposure-induced iBAT activation and iWAT browning. iBAT and iWAT were collected from WT and *Trpm2^−/−^* mice kept at 30°C (black bars), or at 6°C (blue bars). qPCR analyses were performed to measure mRNA levels of: *Pgc-1α* in iBAT **(A)** and iWAT **(B)**; *Ucp1* in iBAT **(C)** and iWAT **(D)**. Results are mean ± SD of determinations on tissues from different animals (n= 4). **(E)**, Western blot analyses were performed in iBAT of WT and *Trpm2^-/-^* mice to evaluate UCP1 protein levels (n= 3): values were normalized on the respective Vinculin levels and mean ± SD of the ratios are shown. **(F)** Sections of paraffin-embedded iBAT and iWAT, processed as described in Material and Methods, were stained with the anti-UCP1 antibody, followed by the anti-rabbit Cy3 secondary antibody. **(G)** Hematoxylin/Eosin (HE) staining was performed on sections of paraffin-embedded tissues using standard procedures. Representative images are shown in F and G. Data were analyzed by ANOVA followed by Tukey’s test: *, p<0.05, **, p<0.01, ***, p<0.001, ****, p<0.0001; ^#^, p<0.05, ^##^, p<0.01, ^###^, p<0.001, compared with the corresponding WT.

To unveil the role of TRPM2 during thermogenesis in adipose tissues, the gene expression levels of *Pgc1-*α and *Ucp1*, two thermogenic markers, were measured in iBAT and iWAT, harvested from WT and *Trpm2^-/-^* mice, housed for 6 days at 22°C and a final day at 6°C, or 7 days at 30°C, as control condition. As expected, an upregulation of these genes was observed in iBAT and iWAT from WT mice exposed to 6°C, in comparison with the control temperature (**Figures 1A-D**). Notably, the cold-induced upregulation of *Pgc1-*α and *Ucp1* levels was significantly attenuated in iWAT and iBAT from *Trpm2^-/-^* mice (**Figures 1A-D**). In agreement with the gene study evaluations, Western blot analyses on iBAT of WT and *Trpm2^-/-^* mice confirmed that a proper *Ucp1* upregulation is hampered in knockout mice exposed to cold (**Figure 1E**). Analyses in immunohistochemistry confirmed a reduced upregulation of UCP1 in both BAT and WAT in *Trpm2^-/-^* mice in response to cold, compared to controls (**Figure 1F**). Importantly, histology showed that BAT activation and the presence of beige cells in WAT are reduced in *Trpm2^-/-^* mice, compared to controls, as revealed by the morphological appearance (including lipid droplet sizes) upon H&E staining (**Figure 1G**).

**Figure 2:**
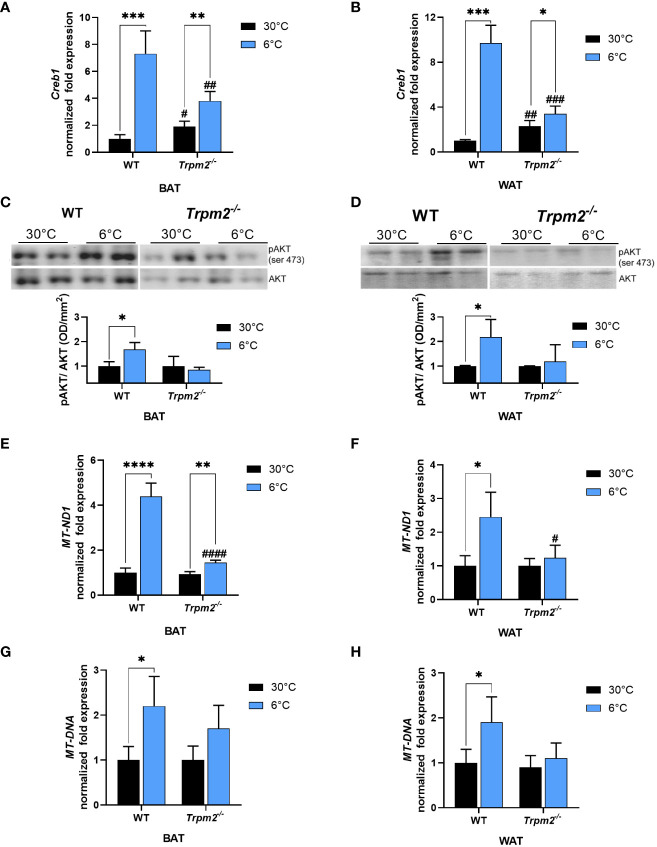
Lack of TRPM2 impairs *Creb1* expression, Akt activation and mitochondriogenesis in response to cold in adipose tissues. iBAT and iWAT were collected from WT and *Trpm2^−/−^* mice kept at 30°C (black bars), or at 6°C (blue bars). qPCR analyses were performed to measure mRNA levels of: *Creb1* in iBAT **(A)** and iWAT **(B)**; *MT-ND1* in iBAT **(E)** and in iWAT **(F)**. The mitochondrial/nuclear DNA ratio was evaluated by qPCR in iBAT **(G)** and iWAT **(H)**. **(C, D)**, Western blot analyses were performed in iBAT and iWAT of WT and *Trpm2^-/-^* mice to evaluate level of phosphorylated Akt (n = 3): values were normalized on the respective Akt levels and mean ± SD of the ratios are shown. Data were by ANOVA followed by Tukey’s test: *, p<0.05, **, p<0.01, ***, p<0.001, ****, p<0.0001; ^#^, p<0.05, ^##^, p<0.01, ^###^, p<0.001, ^####^, p<0.0001, compared with the corresponding WT.

Recently, TRPM2 has been demonstrated to regulate the expression of CREB in an acute myeloid leukemia cell line (39). CREB is a well-known gene master regulator, whose transcription is regulated by Ca^2+^ levels (40), that promotes expression of several genes, including *Pgc1-*α and *Ucp1* (41, 42). Therefore, *Creb1* mRNA levels were evaluated in both iWAT and iBAT of WT and *Trpm2^-/-^* mice. As expected, upon cold exposure *Creb1* expression increased in iWAT (by approximately 7 folds), and in iBAT (by approximately 9 folds) of WT mice, in comparison to mice kept at control temperature (**Figures 2A, B**). On the other hand, *Creb1* mRNA upregulation was greatly reduced in iBAT and iWAT from *Trpm2^-/-^* mice: *Creb1* expression increased only by 2 and 1.5 folds in iBAT and iWAT, respectively, when compared with the corresponding adipose tissues from *Trpm2^-/-^* mice at 30°C (**Figures 2A, B**). As Akt is an important mediator of the cold-induced response, the level of Akt phosphorylation was compared in the different conditions: as shown in **Figures 2C, D**, the cold-induced increase in Akt phosphorylation was abrogated in the absence of TRPM2, in both iBAT and iWAT (**Figures 2C, D**).

Being *Ucp1*, *Pgc1-α* and *Creb1* related to the mitochondrial metabolism, the expression level of *MT-ND1*, the gene encoding for NADH:ubiquinone oxidoreductase core subunit 1, forming the mitochondrial complex 1 in the OXPHOS process, was evaluated: *MT-ND1* gene levels in BAT underwent a 4-fold upregulation upon cold stimulation in WT mice, and only a 1.5-fold upregulation in *Trpm2^-/-^* mice, indicating a reduced OXPHOS function during thermogenesis in mice lacking TRPM2 channel (**Figure 2E**). Regarding *MT-ND1* expression, similar results were obtained in WAT (**Figure 2F**). The mitochondrial/nuclear DNA ratio confirmed that the absence of TRPM2 hampered the increase in mitochondrial content, upon cold exposure in both BAT and WAT (**Figures 2G, H**). Taken together, these data indicate that TRPM2 channels are involved in iWAT browning and in iBAT activation in response to cold.

### 3.2 *Trpm2* expression and ADPR levels are higher in adipose tissue during cold exposure

**Figure 3:**
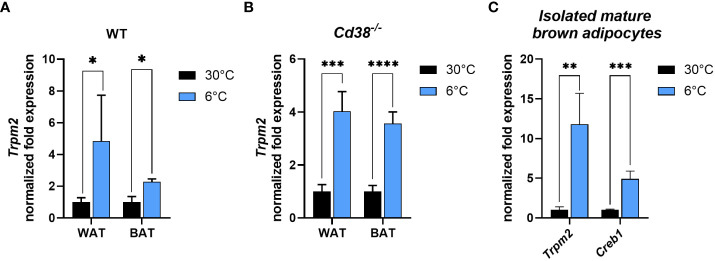
Cold exposure determines the increase in *Trpm2* and *Creb1* in adipocytes. **(A, B)**, qPCR were performed on iBAT and iWAT collected from WT **(A)** and *Cd38^−/−^* **(B)** mice kept at 30°C (black bars), or at 6°C (blue bars) and *Trpm2* mRNA levels were evaluated. **(C)**, *Trpm2* and *Creb1* expression were measured by qPCR on mature brown adipocytes freshly isolated from iBAT of WT mice kept at 30°C (black bars), or at 6°C (blue bars). Results are mean ± SD of determinations on tissues from different animals (n = 4). Data were analyzed by ANOVA followed by Tukey’s test: *, p<0.5, **, p<0.01, ***, p<0.001, ****, p<0.0001.

Since our data suggested an important role for TRPM2 in iWAT browning and iBAT activation (**Figure 1**), *Trpm2* expression was investigated in adipose tissue in response to cold exposure. Importantly, and in line with TRPM2 being pivotal for an appropriate thermogenic response, *Trpm2* was significantly higher in both iBAT and iWAT of WT mice exposed to cold (by 2 and 5 folds, respectively) in comparison with mice housed at control temperature (**Figure 3A**). Recently, we demonstrated that in mice lacking *Cd38*, the induction of thermogenic markers at cold temperature was more pronounced than in WT mice (30). *Trpm2* expression level increased in response to cold in iBAT and iWAT of *Cd38^-/-^* mice (for iBAT even to a greater extent compared to WT mice) (**Figures 3A, B**). To verify whether *Trpm2* expression levels were regulated in adipocytes or in other cell types present in the adipose tissue, mature brown adipocytes were isolated from iBAT harvested from mice exposed to 30°C or 6°C. Similar to the *in vivo* situation, a 12-fold higher *Trpm2* expression was detected in brown adipocytes of cold-exposed compared to warm-housed mice (**Figure 3C**). Likewise, a 5-fold *Creb1* overexpression was detected in these cells (**Figure 3C**).

**Figure 4:**
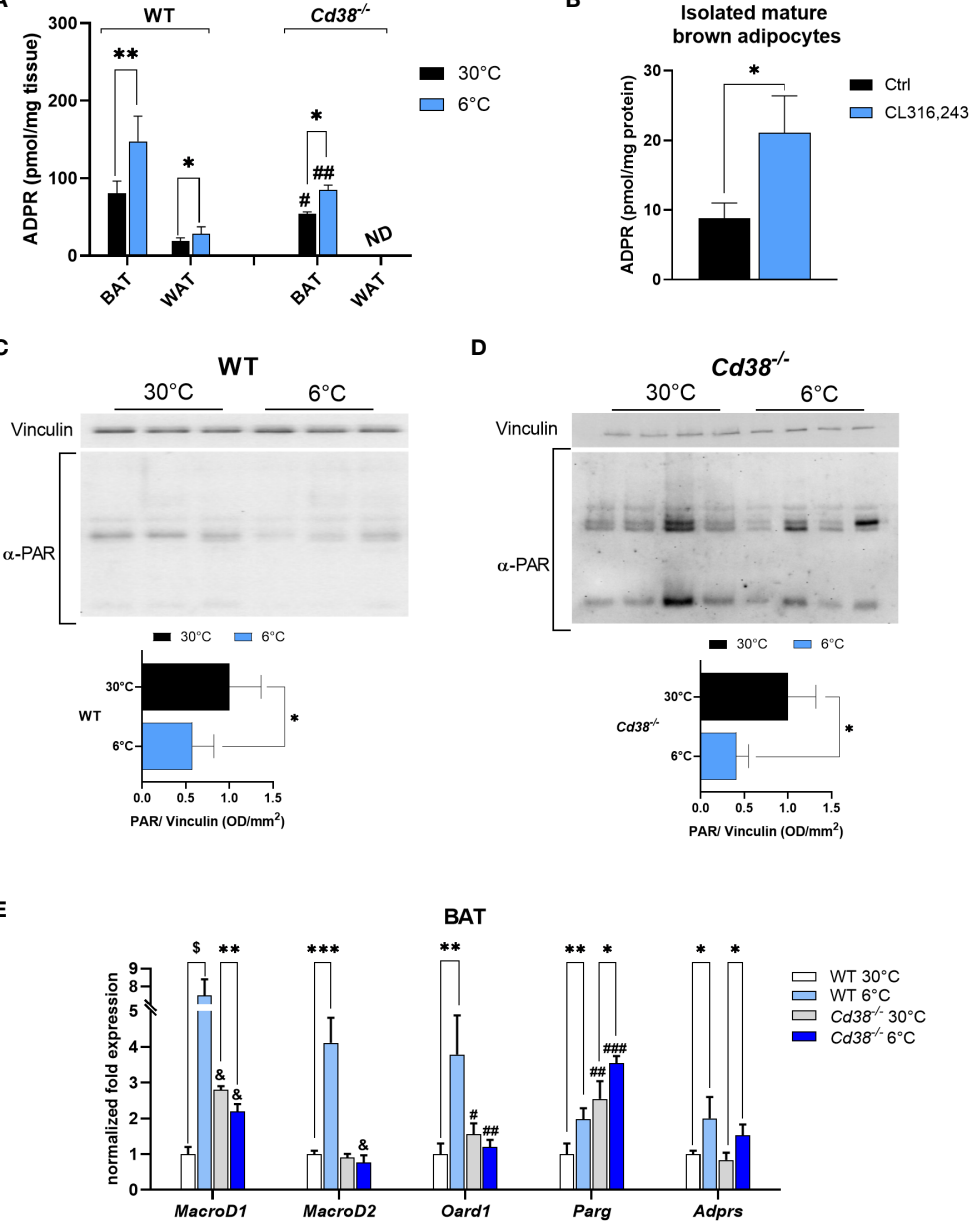
Enhanced degradation of PAR polymers is a source of ADPR during cold exposure in WT and *Cd38^-/-^* mice. ADPR levels were measured by mass spectrometry analyses: **(A)**, on iBAT and iWAT of WT and *Cd38^-/-^* mice kept at thermoneutrality (30°C, black bars), and mice exposed to cold temperature (6°C, blue bars) and results are mean ± SD of determinations on tissues from different animals (n = 6); **(B)** on mature brown adipocytes isolated from WT mice, and exposed to 1 μM CL for 4 h. **(C, D)**, Levels of PARylated proteins revealed by Western blot analyses performed in iBAT of WT **(C)** and *Cd38^-/-^* **(D)** mice housed at 30°C (black bars) or at 6°C (blue bars) using an antibody against ADPR polymers; values were normalized on the respective Vinculin and presented as mean ± SD (n=3). **(E)**, Expression level of the genes encoding for the main cellular mono/poly-ADPR hydrolases (*MacroD1*, *MacroD2*, *Oard1*, *Parg*, *Adprs*) was measured by qPCR on iBAT of WT and *Cd38^-/-^*. Results are mean ± SD of determinations on tissues from different animals (n=4). Data were analyzed by ANOVA followed by Tukey’s test: *, p<0.5, **, p<0.01, ***, p<0.001, $, p<0.00001; #, p<0.05, ##, p<0.01, ###, p<0.001, &, p<0.00001 compared with the corresponding WT.

ADPR is the main TRPM2 agonist produced by NAD^+^ degradation in a number of enzymatic reactions, including those pathways involving CD38, sirtuins and the (poly)ADP-ribosylating/degrading processes (30, 43–45). CD38 is considered the major ADPR-producing enzyme, and its expression and activity are often linked to intracellular Ca^2+^ signaling. CD38 expression is down-regulated in iBAT and iWAT when mice are exposed to cold (30), whereas TRPM2 expression is up-regulated (**Figure 3**). Thus, we evaluated whether ADPR levels are modulated during thermogenesis. To this purpose, mass spectrometry analyses were performed on lysates of iBAT from WT and *Cd38^-/-^* mice exposed to cold or warm ambient temperatures. Despite the fact that CD38 was downregulated during thermogenesis, ADPR levels were approximately 2 fold higher in iBAT from WT mice upon cold exposure (**Figure 4A**). Furthermore, compared to warm housing conditions, ADPR was increased by 1.6 fold even in BAT from cold-exposed *Cd38^-/-^* mice (**Figure 4A**). Nevertheless, basal ADPR levels were significantly lower in *Cd38^-/-^* compared to WT mice (**Figure 4A**), indicating that ADPR is produced by CD38-dependent and independent pathways in BAT in response to cold-induced thermogenesis. ADPR levels were increased from 19 ± 4 to 28 ± 9 pmol/mg tissue in iWAT from mice housed at 30°C or 6°C, respectively (n=6, p<0.05; **Figure 4A**); ADPR levels were not detectable (nd) in WAT from *Cd38^-/-^* mice, suggesting that CD38 has a prominent role in ADPR generation in this tissue (**Figure 4A**). Finally, isolated, mature brown adipocytes were incubated in the presence of 1 μM CL for 4 h: β-adrenergic stimulation determined a significant increase in ADPR content (**Figure 4B**).

### 3.3 Enhanced poly(ADPR) polymer degradation in WAT and BAT of cold-exposed mice

Poly(ADPR) polymerases (PARPs) are enzymes using NAD^+^ to build poly(ADPR) (PAR) polymers on amino acid residues, influencing protein localization and activity. The process of PAR polymer degradation results in a release of ADPR monomers (44, 45). To identify the pathway responsible for the observed ADPR increase (**Figure 4A**), Western blot analyses were performed on iBAT from WT and *Cd38^-/-^* mice, using an antibody against PAR polymers. Interestingly, the amount of PAR polymers was greatly reduced when mice were exposed to cold in comparison with controls (by 50 and 60% in WT and *Cd38^-/-^* mice, respectively; **Figures 4C, D**). Thus, during the thermogenic response, PAR polymers were reduced. Therefore, it is reasonable to suppose that increased ADPR levels in cold-exposed mice are due to an accelerated degradation of PAR polymers.

Next, qPCR analyses were performed to follow the expression of the major (poly)ADP-ribosyl hydrolases, i.e. *MacroD1*, *MacroD2*, *Oard1*, *Parg* and *Adprs* [encoding, respectively, for: MACRO domain-containing protein 1, MACRO domain-containing protein 2, Terminal ADP-ribose protein glycohydrolase 1 (TARG1), Poly(ADP-ribose) glycohydrolase, ADP-ribose glycohydrolase (ARH3)). Gene expression measurements unveiled a marked upregulation of all these enzymes in iBAT, when WT mice were exposed to cold temperature (**Figure 4E**; white vs light blue bars). *Parg* and *Adprs* were also significantly upregulated upon cold exposure in BAT from *Cd38^-/-^* mice (**Figure 4E**; grey vs blue bars). *Parg*, *Oard1* and *MacroD1* were more expressed in iBAT from *Cd38^-/-^* mice compared with WT controls, housed at the thermoneutral temperature (**Figure 4E**; white vs grey bars). Altogether, these data suggest that ADPR may be generated mainly by PARG and ARH3 activities during cold exposure.

### 3.4 Pharmacological inhibition of TRPM2 impairs thermogenic gene expression in white and brown adipocytes

**Figure 5:**
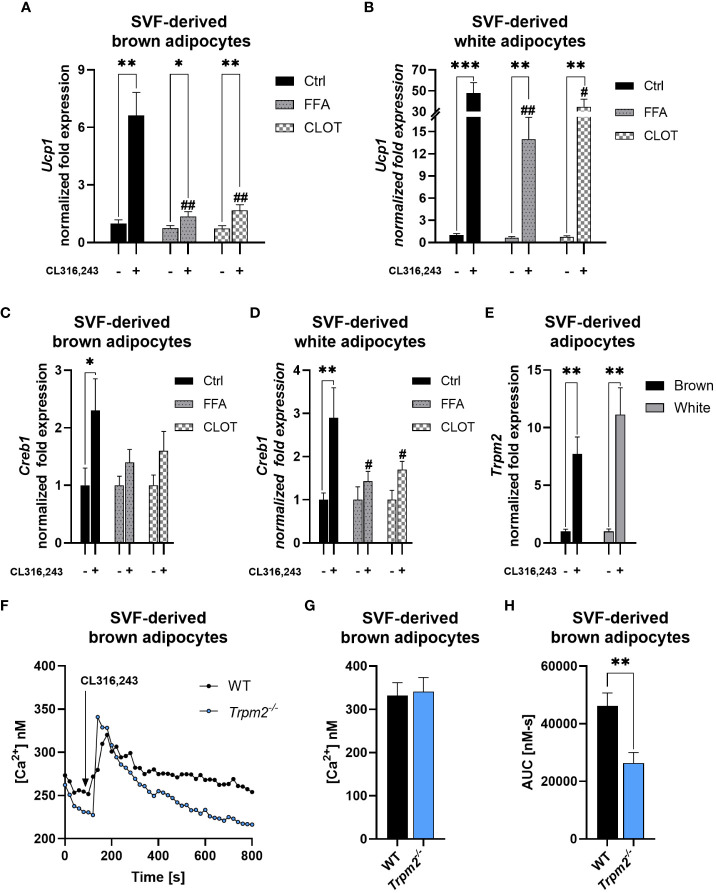
TRPM2 inhibitors hampered thermogenic response in CL316,243-treated primary adipocytes. SVF-derived cells were isolated from iBAT and iWAT and differentiated using a specific differentiation cocktail: mature adipocytes were stimulated (or not) with 100 nM CL316,243, in presence or absence of TRPM2 inhibitors. **(A-D)**, qPCR analyses of *Ucp1* **(A, B)** and *Creb1* **(C, D)** expression in brown **(A, C)** and white **(B, D)** primary adipocytes pre-treated with 200 μM flufenamic acid (FFA), 100 μM clotrimazole (CLOT) or vehicle, prior to CL treatment. **(E)**, *Trpm2* expression in white and brown adipocytes upon CL treatment. Results are mean ± SD of at least 4 determinations. **(F-H)**, SVF-derived brown adipocytes from WT and *Trpm2* ^-/-^ mice were loaded with Fura2, rinsed twice and a Ca^2+^-containing buffer was added. The Fura2-loaded adipocytes were imaged on a Leica IRBE microscope and stimulated with 1 µM CL. The mean trace **(F)** is shown (n=49 for WT, n = 40 *Trpm2* ^-/-^), together with the calculated mean peak **(G)** and the area under the curve **(H)**. Data were analyzed by ANOVA followed by Tukey’s test: *, p<0.5, **, p<0.01, ***, p<0.001; #, p<0.05, ##, p<0.01 compared with the corresponding WT.

To evaluate the impact of TRPM2 pharmacological inhibition on thermogenic adipocytes in a cell-autonomous manner, stromal vascular fraction (SVF)-derived cells were collected from iBAT and iWAT of WT mice and then differentiated to mature adipocytes. Then, adipocytes were activated using the β-3-adrenergic receptor agonist CL. To assess whether the impaired expression of thermogenic genes observed in *Trpm2*-deficient mice is ascribable to adipocyte dysfunctions, FFA and CLOT, two known TRPM2 inhibitors (46) were added to differentiated cells prior to CL treatment. *Ucp1* expression levels were increased in brown and white adipocytes in response to CL treatment (**Figures 5A, B**), as expected. However, *Ucp1* induction was substantially lower in adipocytes pretreated with TRPM2 inhibitors. In brown adipocytes, *Ucp1* induction was attenuated by approximately 80 and 74% in FFA- or CLOT-pre-treated cells, respectively; in white adipocytes, *Ucp1* expression was reduced by approximately 71 and 27% in FFA- or CLOT-pre-treated cells compared to control conditions, respectively (**Figures 5A, B**). Similar results as for *Ucp1* were obtained by measuring *Creb1* expression (**Figures 5C, D**). Moreover, in line with the *Trpm2* induction in cold-exposed WT mice (**Figure 3**), CL treatment caused a marked upregulation of *Trpm2* expression in both brown and white adipocytes (by 7.7 and 11.1 folds, respectively; **Figure 5E**). Changes in the [Ca^2+^]_i_ were compared in brown adipocytes, differentiated from the SVF obtained from WT or *Trpm2^-/-^* mice: the global CL-induced Ca^2+^ response was reduced in *Trpm2^-/-^* cells (**Figures 5F, H**), although the first peak of Ca^2+^ increase was not significantly different (**Figures 5F, G**). These data further confirm that TRPM2 must be considered of pivotal relevance during the thermogenic response in adipose tissues.

### 3.5 *Trpm2* deletion does not affect the hepatic glycolysis/glucose release regulation during thermogenesis

**Figure 6:**
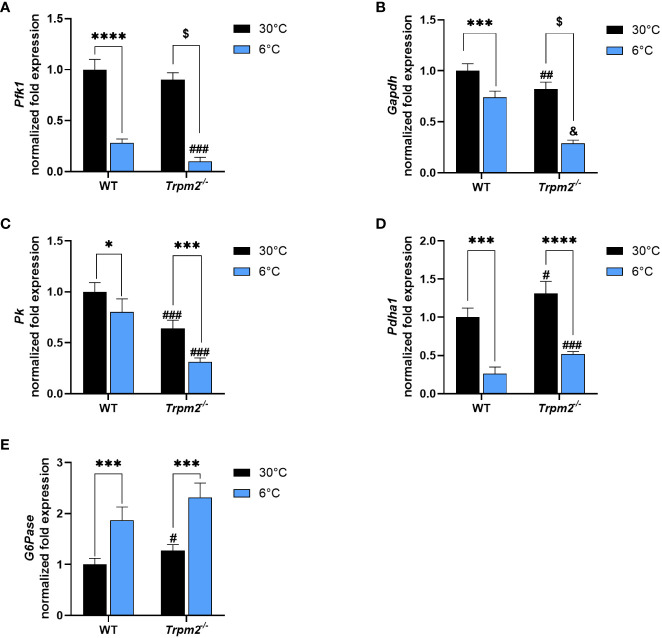
Trpm2 is dispensable in the downregulation of glycolysis and in the upregulation of glucose release occurring in liver upon cold-exposure. Livers were collected from WT and *Trpm2^-/-^* mice kept at 30°C (black bars) or 6°C (blue bars). Phosphofructokinase 1 (*Pfk1*, **(A)**, Glyceraldehyde-3-Phosphate Dehydrogenase (*Gapdh*, **(B)**, Pyruvate Kinase (*Pk*, **(C)**, Pyruvate dehydrogenase (*Pdha1*, **(D)** and Glucose 6-Phosphatase (*G6pase*, **(E)** gene expression in WT and *Trpm2^-/-^* mice. Results are mean ± SD of determinations on tissues from different animals (n = 4). Data were analyzed by ANOVA followed by Tukey’s test: ^*^, p<0.5, ^***^, p<0.001, ^****^, p<0.0001, ^$^, p<0.00001; ^#^, p<0.05, ^##^, p<0.01, ^###^, p<0.001, ^&^, p<0.00001 compared with the corresponding WT.

Liver, as BAT and WAT, is a relevant organ governing thermogenesis, both by providing energy sources and by releasing stimulatory factors that affect peripheral tissues (33). We recently demonstrated that in cold-exposed *Cd38^-/-^* mice, the hepatic cross-regulation between glycolysis and glucose release was lost (33). To investigate whether TRPM2 is relevant in this process, the impact of *Trpm2* depletion on hepatic metabolism during thermogenesis was evaluated. Glycolytic genes (*Pfk1*, *Gapdh, Pk1*, encoding respectively for Phosphofructokinase 1, Glyceraldehyde-3-Phosphate Dehydrogenase and Pyruvate Kinase) and Pyruvate Dehydrogenase *(Pdha1)* expression was down-regulated in WT mice exposed to cold in comparison with WT mice housed at control temperature (**Figures 6A–D**). On the other hand, hepatic glucose release to maintain systemic glucose homeostasis is increased in response to cold exposure, as indicated by higher Glucose 6-Phosphatase (*G6pase)* expression in cold- versus warm housed mice (**Figure 6E**). Notably, *Trpm2* ablation did not seem to affect the pattern of glycolysis/glucose release regulation in liver (**Figure 6**).

### 3.6 *Trpm2* expression and PAR polymer degradation occur also in liver and *Fgf21* expression is affected by *Trpm2* ablation during cold exposure

**Figure 7:**
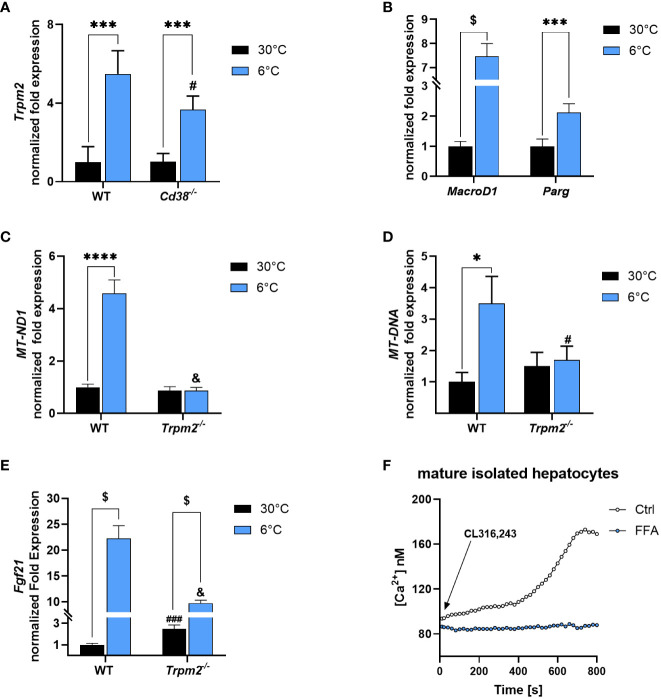
Trpm2 in liver during thermogenesis. Livers were collected from WT, *Cd38^−/−^* and *Trpm2^-/-^* mice kept at 30°C (black bars) or 6°C (blue bars). **(A)**, *Trpm2* gene expression was evaluated in WT and *Cd38^−/−^* by qPCR. **(B, C, E)**, *MacroD1* and *Parg* **(B)**, *MT-ND1* **(C)** and Fibroblast growth factor 21 (*Fgf21*, E*)* gene expression was evaluated in WT and *Trpm2^-/-^* mice by qPCR. **(D)** The mitochondrial/nuclear DNA ratio was evaluated by qPCR. **(F)** Hepatocytes were isolated from WT mice and loaded with Fura2. Cells were pre-incubated (or not) for 10 min with 200 μM FFA and [Ca^2+^]_i_ measurements, upon 1 μM CL316,243 stimulation, were performed with a microfluorimetric system (Cairn Research, Faversham, Kent, UK). Results are mean ± SD of determinations on tissues from different animals (n = 4). Data analyzed by ANOVA followed by Tukey’s test: ^*^, p<0.5, ^***^, p<0.001, ^****^, p<0.0001, ^$^, p<0.00001; ^#^, p<0.05, ^###^, p<0.0001, ^&^, p<0.00001 compared with the corresponding WT.

*Trpm2* expression was measured by qPCR in liver of WT and *Cd38^-/-^* mice housed at 30 or at 6°C. Interestingly, a 5.5- and a 3.7-fold higher *Trpm2* expression was detected in livers of cold-exposed WT and *Cd38^-/-^* mice, respectively (**Figure 7A**). Moreover, hepatic *MacroD1* and *Parg* gene expression were upregulated in WT mice exposed to cold, approximately by 7 and 2 folds, respectively, in comparison to control mice (**Figure 7B**). Thus, the hepatic regulation of *Trpm2*, *MacroD1* and *Parg* expression is similar to that occurring in iBAT.

The absence of *Trpm2* hampered the promotion of mitochondrial metabolism occurring during thermogenesis (4.6-fold higher *MT-ND1* expression, the gene encoding for NADH : Ubiquinone Oxidoreductase Core Subunit 1, in WT mice exposed to 6°C compared to control mice; **Figure 7C**). Moreover, the mitochondrial/nuclear DNA ratio confirmed that, also in liver, the absence of TRPM2 hampered the increase in mitochondrial content, upon cold exposure (**Figure 7D**). Hepatic *Fgf21* gene expression was increased in WT mice exposed to cold (by 22 times). However, this effect was greatly attenuated in liver of *Trpm2^-/-^* mice exposed to cold (**Figure 7E**). Overall, these data suggest that mitochondrial activity in response to cold exposure and the production of FGF21, processes that could contribute to systemic energy expenditure and heat production, may be impaired in TRPM2-deficient mice. Finally, the use of the TRPM2 inhibitor FFA reduced the Ca^2+^ increase induced by CL in isolated hepatocytes (**Figure 7F**), in line with previous reports demonstrating that, in hepatocytes, the epinephrine-induced Ca^2+^ rise was abolished by EGTA (chelating the extracellular Ca^2+^) and by 2-APB (47), which has been reported to act as a TRPM2 antagonist (48). The steady and slow Ca^2+^ increase induced by CL in hepatocytes was very different than the Ca^2+^ rise observed in adipocytes (**Figure 5F**): this is possibly due to the different contribution of the various components determining to the global Ca^2+^ rise (49). Nevertheless, it is noteworthy to remark that the role for TRPM2 in the CL-induced Ca^2+^ increase seems to be more relevant in a “not rapid” phase.

### 3.7 *Trpm2^-/-^* mice exhibit lower respiration and energy expenditure

**Figure 8:**
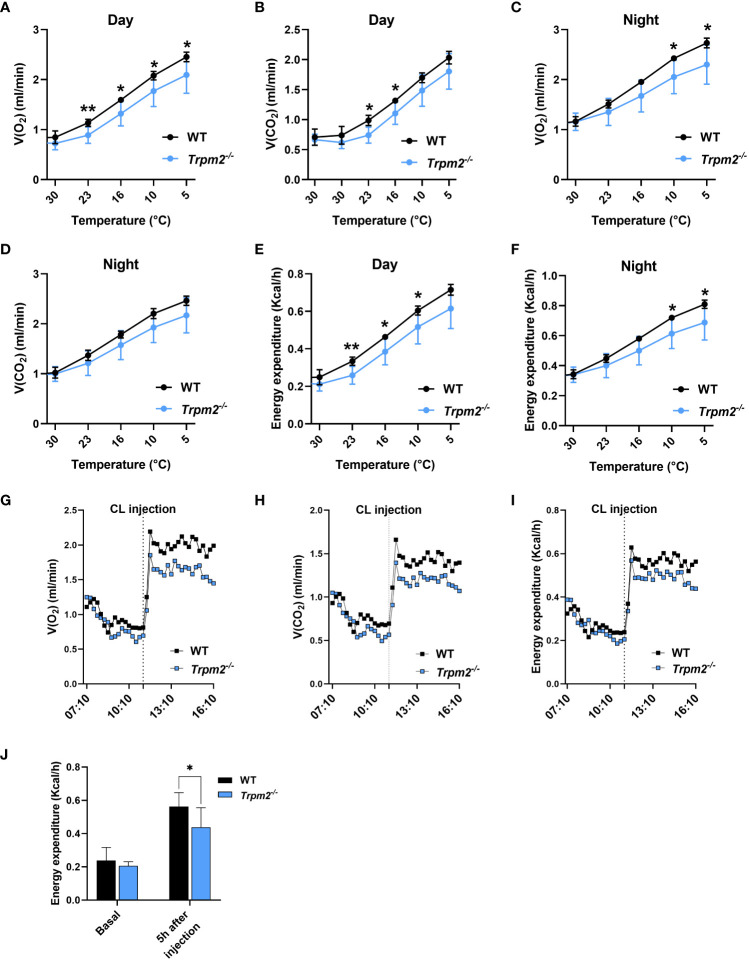
*Trpm2* deficiency reduced the thermogenic capacity in response to cold acclimation and adrenergic stimulation. **(A-D)**, *In vivo* measurements (O_2_ consumption and CO_2_ production) performed on WT (black lines) and *Trpm2^-/-^* mice (blue lines) housed for 5 days to decreasing temperatures (from 30°C to 5°C) during the light time (Day; **(A, B)** and the dark time (Night; **(C, D)**; mean ± SD (n=6 mice per group). **(E, F)**, calculated EE at Day **(E)** and Night **(F)**; mean ± SD (n=6 mice per group). **(G)**, O_2_ consumption, **(H)**, CO_2_ production, and **(I)**, EE in WT (black lines) and *Trpm2^-/-^* mice (blue lines) mice housed at thermoneutrality upon CL administration. Monitoring started approximately 4 h before the treatment and stopped 5 h after the injection of CL; mean traces from n=6 mice per group are shown. **(J)**, EE: mean ± SD (n=6) of calculated values at 1 h before and 5 h after CL injection in WT (black bars) and *Trpm2^-/-^* mice (blue bars). * p ≤ 0.05 and ** p ≤ 0.01 by two-tailed unpaired Student’s t test.

To investigate *in vivo* the consequences of *Trpm2* ablation in mice during cold exposure and adrenergic signaling, two sets of experiments were performed using indirect calorimetry to monitor O_2_ consumption and CO_2_ production. These values were used to calculate energy expenditure (EE) in WT and TRPM2-deficient mice in a so-called Scholander experiment, where a stepwise decrease in the ambient temperature results in increased EE to maintain a constant body core temperature. As expected, both O_2_ consumption and CO_2_ production increased at day (**Figures 8A, B**) and at night (**Figures 8C, D**). In line with these data, a substantial increase in EE was observed when the ambient temperature was reduced at daytime (**Figure 8E**) and at night (**Figure 8F**). In *Trpm2^-/-^* mice, O_2_ consumption and by trend CO_2_ production were lower at colder temperatures, which compared to WT resulting in significantly lower EE. Next, to investigate the total thermogenic capacity of BAT, mice housed at thermoneutrality were injected with CL to specifically activate thermogenesis in BAT (**Figures 8G-J**). As expected, CL treatment caused a substantial increase in O_2_ consumption, CO_2_ production and EE in mice (**Figures 8G-J**). Notably, *Trpm2^-/-^* mice exhibited attenuated thermogenic response when compared to WT mice, exhibiting lower O_2_ consumption, CO_2_ production and EE in comparison with WT treated mice (**Figures 8G-J**). Overall, these data indicate that TRPM2 plays an important role in adaptive thermogenesis and energy homeostasis in response to cold and β3-adrenergic receptor activation.

## 4 Discussion

The aim of the present study was to evaluate the role of TRPM2 in WAT, BAT and liver during thermogenesis. We had previously demonstrated that the expression of CD38 is downregulated in response to cold exposure, which modulates the content of NAD(P)(H) in adipose tissues (30). CD38 and TRPM2 are usually considered as sequentially linked in a shared signaling axis, impacting on the intracellular levels of the third messenger Ca^2+^ as CD38 generates ADPR, the main TRPM2 agonist.

The role of various TRPs channels in adipose tissue metabolism has been extensively studied in the last two decades. TRPV1 is a calcium channel activated by capsaicin and, when activated by agonists, prevents obesity induced by HFD: the effect was abolished in mice lacking this channel (50). In addition, dietary TRPV1 activators are involved in adrenaline secretion from the nervous system, thus enhancing WAT browning and in turn, energy expenditure (51). On the contrary, TRPV4 seems to play a negative role in thermogenesis. Indeed, the expression of the browning markers PGC1-α and UCP1 is promoted upon TRPV4 suppression (52). In addition, mice lacking TRPV4 are resistant to HFD-induced obesity (53). The most studied TRP channel in the context of adipose tissue biology and thermogenesis is TRPM8. As a thermoreceptor, this channel is activated not only by cold-mimicking compounds (such as menthol and icilin), but also by cold temperature (54). Different studies reported that TRPM8 activation through menthol administration *in vitro* enhances UCP1 expression, mitochondrial activation and heat production, and thus promotes WAT browning and BAT activation (55–59). Furthermore, *in vivo* dietary menthol supplementation reduces insulin resistance and ameliorates HFD-induced obesity (55, 57).

Mice lacking *Cd38* exhibit enhanced thermogenesis, as measured by the expression of the two main browning markers (30). Through the use of mice lacking *Trpm2*, we thus aimed at clarifying whether the results obtained from *Cd38^-/-^* mice in adipose tissue are a consequence, not only of an altered NAD^+^ consumption, but also of a modified activation of the TRPM2 channel. If CD38 effect were due to the lack of ADPR for TRPM2 gating, it would be reasonable to hypothesize that ADPR-induced TRPM2 signaling could be detrimental for thermogenesis in adipose tissue. Instead, the obtained data unequivocally demonstrate that the absence or inhibition of TRPM2 significantly reduce the response to thermogenic stimuli. Indeed, mice lacking *Trpm2* exhibit lower respiration rate and reduced energy expenditure when thermogenesis is stimulated both by cold temperature and by β3-adrenergic receptor activation (**Figure 8**). In addition, gene expression analyses confirmed that the browning process is reduced in WAT and BAT (**Figure 1**) in *Trpm2^-/-^* mice upon cold exposure. Finally, TRPM2 pharmacological inhibition hampered *Ucp1* expression in white and brown primary adipocytes (**Figures 5A, B**).

Furthermore, the increased expression of *Trpm2* in WAT and BAT (specifically in brown adipocytes within BAT) upon short-term cold exposure (**Figures 3A-C**), and in primary adipocytes stimulated with CL (**Figure 5E**) confirm that TRPM2 plays a pivotal role in thermogenesis. Notably, *Trpm2* induction also occurs in mice lacking *Cd38*, at similar levels in WAT, and even at higher levels in BAT (**Figures 3A, B**), suggesting that: a) the *Trpm2* transcription occurs independently of CD38-mediated signaling pathways; b) a higher expression of *Trpm2* may be associated to an improved BAT activation, as occurring in *Cd38^-/-^* mice (30). Admittedly, the exact mechanism regulating Trpm2 transcription has not been defined and will represent an interesting new area of investigation.

As mentioned above, TRPM2 activation is considered to be due to the CD38-mediated production of ADPR. Instead, in thermogenesis, CD38 and TRPM2 are regulated in opposite directions: *Trpm2* is overexpressed (**Figure 3**), whereas CD38 is downregulated (30). Being CD38 one of the major ADPR producers, its reduced expression could be associated with reduced ADPR levels and, as a consequence, lower TRPM2 activation. Instead, we observed that ADPR levels rise in WAT and BAT (**Figure 4A**) when WT mice are exposed to cold, i.e. when CD38 is down-regulated (30). Moreover, ADPR levels were increased also in BAT of *Cd38^-/-^* mice (**Figure 4A**), in line with the conclusion that CD38 cannot represent the source of ADPR during cold exposure, and that the CD38-mediated ADPR production does not impact BAT activation. In this respect, a possible detrimental role for cyclic ADP-ribose (activating ryanodine receptors), which would not be produced in the absence/downregulation of CD38, cannot be ruled out at present. However, an enhanced Ca^2+^ cycling, upon activation of α1/β3-adrenergic receptors or the SERCA2b-ryanodine receptor 2 pathway, stimulates UCP1-independent thermogenesis in beige adipocytes (60), suggesting that reduced production of cyclic ADP-ribose is unlikely to represent the reason for an improved thermogenesis in *Cd38^-/-^* mice (30).

Altogether, our findings unveil for the first time that ADPR is likely pivotal in the activation of the thermogenic program in WAT and BAT, possibly by regulating cytosolic Ca^2+^ levels (**Figure 5F**), and that enzymes other than CD38 are responsible for ADPR generation in thermogenic adipose tissues in response to cold exposure. The hydrolysis of mono-ADP-ribosyl (MAR) and/or poly-ADP-ribosyl groups may represent a pathway that provides free ADPR to sustain TRPM2 activation (**Figure 4**). Our data suggest that PAR hydrolysis could contribute to ADPR generation during cold exposure, since: a) the expression of *Parg* and *Adprs* (acting on PAR) is up-regulated also in *Cd38^-/-^* mice (**Figure 4E**); b) *Parg* is one of the most expressed enzymes among the MAR and PAR hydrolases tested; c) the level of PARylated proteins is significantly reduced upon cold exposure (**Figures 4C, D**).

Previous studies reported alternative TRPM2 activation pathways that do not involve CD38 activity: 2’-O-Acetyl-ADPR (OAADPR) generated by SIRT2 and SIRT3 is able to mediate TRPM2 gating (61); OAADPR and/or ADPR produced by SIRT6 regulates TRPM2 gating that, in turn, promotes the release of pro-inflammatory cytokines and pancreatic cell migration (43). Park and colleagues suggested the role of PARPs in TRPM2 activation during high oxidative stress and investigated this mechanism in the context of Alzheimer disease. They showed that amyloid-beta-induced oxidative stress determines a neurovascular dysfunction through TRPM2 overactivation, suggesting that TRPM2 inhibition could be a therapeutic target for this neurodegenerative disease (62).

Regarding the role of PARPs in adipose tissue, PARP1 and 2 inhibition showed a positive effect on energy metabolism, being PARP1 suppression associated to higher SIRT1 activity, leading to higher energy expenditure and increased mitochondrial activity (63). In line with this, *Parp2* genetic ablation increased energy expenditure in mice and promoted mitochondrial biogenesis in different tissues, including liver. This is due to the negative effect exerted by PARP2 on *Sirt1* promoters, that suppress its expression (64).

To the best of our knowledge, our study is the first one reporting an induction of PAR, as well as MAR hydrolases, in association to WAT browning and BAT activation, revealing ADPR increase and higher TRPM2 expression in response to cold. It is of note that CD38 plays a crucial role in the cross-regulation of glycolysis and gluconeogenesis in liver in response to cold-induced thermogenesis (33), which is in line with the importance of CD38-mediated cADPR production and Ca^2+^ signaling in glucagon-induced stimulation of gluconeogenesis (65). Conversely, the ADPR-TRPM2 axis does not seem to be relevant in the regulation of glycolysis and gluconeogenesis, at least during thermogenesis, since *Trpm2^-/-^* ablation does not affect the down-regulation of glycolytic genes and the upregulation of G6P phosphatase (**Figure 6**). Instead, the lack of *Trpm2* seems to impair mitochondrial function and reduce FGF21 secretion by the liver (**Figures 7C-E**), which both could contribute to the systemic hampered thermogenic response. Importantly, it has been recently demonstrated that the β-adrenergic-dependent increase in circulating FGF21 (which derives mainly from liver) occurs through an indirect mechanism: adipocyte-derived fatty acids stimulate FGF21 expression in liver (66). Thus, the reduced levels of FGF21 in *Trpm2^-/-^* mice is likely a consequence of the hampered response to cold in adipose tissue, although a role for hepatic TRPM2 in mediating *Fgf21* production cannot be excluded.

Overall, in adipocytes, TRPM2 may be activated downstream of β-adrenergic receptors’ stimulation, through multiple pathways: a) increased ADPR content, as a consequence of increased PAR degradation (**Figure 4**); b) cAMP increase, which-may activate TRPM2 both through the activation of PKA and/or EPAC, as reported to occur in other cell types (67, 68); c) increased mitochondrial activity in BAT during cold exposure is accompanied by the production of ROS (69), known TRPM2 agonists (17, 18).

Our data indicate that TRPM2 activation regulates CREB expression level and Akt phosphorylation: a) the increase in *CREB* levels were lower in *Trpm2^-/-^* BAT and WAT from mice exposed to cold and in isolated mature brown adipocytes collected from mice exposed to cold (**Figures 2A, B**); b) the CL-induced increase in *CREB* expression was reduced in SVF-derived adipocytes upon TRPM2 pharmacological inhibition (**Figures 5C, D**); c) in *Trpm2^-/-^* BAT and WAT, the cold-induced increase in phosphorylation of Akt was reduced (**Figures 2C, D**). CREB has been reported to be downstream of TRPM2 activation in neuroblastoma cells (70) and in AML cells (39). In addition, the fact that CREB expression is downstream of Ca^2+^ increase has been well documented (40). Akt activation is known to lie downstream of TRPM2 activation in different models, including gastric cancer cell and aortic smooth muscle cells (71, 72).

Intriguingly, TRPM2 has been recently demonstrated to play an important role in body temperature regulation acting as a temperature sensor in a subpopulation of hypothalamic neurons (73) and the homeostatic body-response to warm temperature was hampered in *Trpm2^-/-^* mice (74). From the result presented in our study, a role for hypothalamic TRPM2 in the detection of cold temperatures cannot be ruled out. Nevertheless, in Brandt’s voles, TRPM2 expression was strongly up-regulated in BAT upon cold-exposure, in line with our findings, whereas TRPM2 expression was not modified in the hypothalamus (75).

In conclusion, our results demonstrate that increased NAD^+^ levels, obtained through physiological downregulation of CD38 in response to cold (as during thermogenesis; 30, 33), or following CD38 pharmacological inhibition, are not impacting TRPM2 activity through a hampered CD38-dependent production of ADPR. This is of great interest in the field of chronic inflammatory diseases, given the numerous efforts of the research community to increase intracellular NAD^+^ levels for the treatment of various pathological conditions. The fact that cold-induced thermogenesis leads to a lower NAD^+^ degradation in adipose tissue and liver may uncover a further benefit of this approach, which is under investigation for the amelioration of several metabolic disturbances. Moreover, since *Cd38^-/-^* mice are able to sustain an enhanced BAT activation and WAT browning upon cold stimulation, NAD^+^ availability seems to be a factor limiting these processes. Indeed, the phenotype we observed in *Cd38^-/-^* mice upon cold stimulation, is not to be ascribed to the impaired activation of TRPM2 channel, since the lack of TRPM2 leads to a reduced WAT browning and BAT activation.

Further studies are required to investigate whether pharmacological TRPM2 activation may represent a new therapeutic strategy for the treatment of metabolic diseases, aimed at improving thermogenic capacity and activity of WAT and BAT.

## Data availability statement

The datasets presented in this study can be found in online repositories. The names of the repository/repositories and accession number(s) can be found below: not applicable.

## Ethics statement

The animal studies were approved by Animal Welfare Officers at University Medical Center Hamburg-Eppendorf. The studies were conducted in accordance with the local legislation and institutional requirements. Written informed consent was obtained from the owners for the participation of their animals in this study.

## Author contributions

Conceptualization, SB, AG, and JH. Conception and design of the study, AB, MH, JH, SB. Methodology and Investigation, AB, MH, SS, AS, AW, BD, CA, RPM, AM, MJ, VV. Resources, FK-N and H-WM. Data Curation, AB and SB. Writing Original Draft Preparation, AB and SB. Writing Review and Editing, SB, H-WM, AF, JH. Supervision, LS, GD, JH, and SB. Funding Acquisition, FK-N, H-WM, AG, JH, and SB. All authors contributed to the article and approved the submitted version.
